# The impact of simulation-based emergency training on novice critical care nurses: a quasi-experimental study

**DOI:** 10.5116/ijme.68a2.dc6f

**Published:** 2025-08-29

**Authors:** Huang Yi-Chen, Lin Chair-Hua, Yao Ting-Yu, Lee Tsung-Han, Chiang Tsay-I, Lin Chih Hao

**Affiliations:** 1 Department of Nursing, Changhua Christian Hospital, Taiwan; 2 Department of Emergency Medicine, Changhua Christian Hospital, Taiwan; 3 Department of Nursing, Hung Kuang University, Taiwan; 4 Department of Big Data and Digital Promotion Center, Changhua Christian Hospital, Taiwan

**Keywords:** Simulation-based training, emergency care, nursing education, critical care nurses, novice nurses

## Abstract

**Objectives:**

This quasi-experimental study evaluated the impact of simulation-based emergency training on novice critical care nurses’ knowledge, skills, and confidence compared to traditional clinical teaching, aiming to enhance their preparedness for high-pressure emergency scenarios.

**Methods:**

A pretest-posttest non-equivalent control group design was conducted in a Taiwan medical center’s critical care unit from October 2023 to January 2024. Sixty-seven nurses with less than two years of experience were recruited via convenience sampling and assigned to an experimental group (n=32, simulation-based training) or a control group (n=35, traditional teaching). The experimental group underwent an OSCE-based intervention with three stations. Data were collected using the Nursing Competency Questionnaire, OSCE scoring rubric, and Learning Satisfaction Scale. Paired and independent t-tests with effect sizes (Cohen’s d) were used for analysis.

**Results:**

The simulation group showed significant improvements in skills (t_(31) _= 1.92, p = .016, d = .34) and confidence (t_(31)_ = 2.92, p = .004, d = .40); the traditional group improved in confidence only (t_(34)_ = 2.24, p = .027, d = .33). No significant between-group differences were found (e.g., skills: t_(65)_ = 1.29, p = .201, d = .33).

**Conclusions:**

Simulation-based training effectively enhances skills and confidence in novice critical care nurses, complementing traditional methods. Integrating both approaches can optimize training outcomes, improving patient safety and nurse retention in medical education. These findings advocate for incorporating simulation into nursing curricula to better prepare novice nurses for emergency care. Future research should explore multi-center studies with objective measures.

## Introduction

Simulation-based emergency training has become a vital tool for enhancing the confidence and preparedness of novice critical care nurses with less than two years of clinical experience.^1^ Emergency care is a common yet critical clinical scenario requiring rapid assessment and timely intervention. However, newly recruited nurses often lack the experience and confidence necessary to effectively manage deteriorating patients.^2^ This gap can result in uncertainty, hesitation, and frustration, compromising patient safety during emergencies.^3^^-^^5^

Research indicates that when nurses encounter acute patient deterioration, each second counts. Accurate and timely interventions, supported by effective teamwork, are essential to ensure optimal patient outcomes. Without adequate training, novice nurses may struggle to meet the demands of high-pressure emergency situations, negatively impacting the quality of care.^6^^-^^7^ Although existing studies have extensively explored simulation-based training in nursing education, they predominantly focus on nursing students or nurses in general wards and emergency departments. Limited research has specifically addressed the needs of novice critical care nurses during their early career, a population facing unique challenges due to the high-stakes nature of their work.8 This study fills this gap by examining how simulation-based training, using the Objective Structured Clinical Examination (OSCE) method, bridges theoretical knowledge and practical skills in this understudied group.

Traditional assessments, such as written exams, effectively measure cognitive knowledge but do not adequately evaluate practical skills or prepare nurses for real-life clinical scenarios. In contrast, simulation-based training methods, such as the Formative OSCE, integrate real-case scenarios to provide participants with a safe and controlled environment to practice emergency care. This approach fosters the development of essential competencies, including clinical judgment, technical proficiency, and teamwork, while also enhancing learning motivation through structured, hands-on, goal-oriented scenarios.^9^

To meet the demands of clinical practice, it is imperative to assess new nurses’ ability to respond effectively in emergencies.^10^^-^^12^ Simulation-based OSCE training allows nurses to hone their skills under realistic high-pressure conditions, ensuring they can handle critical patients with confidence and competence. Novice nurses can prevent complications and improve patient outcomes by identifying at-risk patients and intervening early.

This study aimed to evaluate the impact of simulation-based emergency training on the emergency knowledge, skills, and confidence of critical care nurses with less than two years of experience. Using the OSCE method, we sought to provide evidence of how simulation-based training can bridge the gap between theoretical knowledge and practical applications, ultimately fostering professional growth and improving retention rates in critical care nursing.

## Methods

### Study design

This study employed a quasi-experimental pretest-posttest non-equivalent control group design, conducted between October 2023 and January 2024, to evaluate the effectiveness of an OSCE-based simulation training on the emergency skills, knowledge, and confidence of novice critical care nurses. This design was chosen due to logistical constraints in a clinical setting, where true randomization could disrupt workflow. Baseline homogeneity and pre/post-test comparisons were used to mitigate threats to internal validity, such as selection bias. The training was designed based on the NLN Jeffries Simulation Theory,^13^^-^^14^ a framework emphasizing simulation as an interactive, learner-centered approach that promotes meaningful engagement, realistic scenario design, and measurable learning outcomes.^9^^-^^10^

### Participants and setting

Seventy critical care nurses with less than two years of clinical experience were recruited from the emergency department and intensive care units of Changhua Christian Hospital, a medical center in central Taiwan, using convenience sampling. A total of 82 nurses were initially screened for eligibility. The inclusion criteria were registered nurses who had completed their probation period, had less than two years of clinical experience in emergency or intensive care settings, and consented to participate. Nurses still under probation or with more than 23 months of experience were excluded, as their proximity to the two-year experience threshold could confound results. Based on these criteria, 12 nurses were excluded, leaving 70 eligible participants. Participants were recruited through open invitations distributed via the hospital’s internal communication system, with the principal investigator providing study details to head nurses, who facilitated communication with their staff. Interested nurses voluntarily registered with their unit head. Participants were sequentially listed, and every second nurse was selected for the experimental group (n = 35), while the remaining nurses were assigned to the control group. The systematic assignment using a fixed interval method aimed to reduce selection bias. The sample size was determined by the number of available novice nurses during the study period, though this limited the study’s statistical power. During the study, three participants in the experimental group left the work environment due to work schedule changes, resulting in a final sample of 32 in the experimental group and 35 in the control group.

Ethical considerations were prioritized throughout the study to protect participants’ rights and well-being. Informed consent was obtained from all participants, who were fully informed about the study’s purpose, procedures, potential risks, and benefits. Participants were assured of their right to withdraw at any time without consequences to their employment or professional standing. To ensure confidentiality, all data were anonymized using unique identifiers, and personal information was stored securely in a password-protected database accessible only to the research team. No invasive procedures or high-risk activities were involved, and the simulation-based training was conducted in a controlled, safe environment. The study posed minimal risk, as it focused on educational interventions, and no adverse events were reported. The Institutional Review Board of Changhua Christian Hospital (IRB No. 230613) reviewed and approved the study protocol, ensuring compliance with ethical standards for human research.

### Instruments

Primary outcomes were emergency clinical skills and confidence, with emergency care knowledge as a secondary outcome. The following tools were used:

#### Emergency Knowledge Questionnaire

The emergency knowledge questionnaire assessed participants’ understanding of basic emergency care principles and OSCE concepts, covering topics such as fundamental resuscitation knowledge and the theoretical basis of OSCE assessments.

#### Nursing Competency Questionnaire (NCQ)

A self-designed tool with 22 items rated on a 5-point Likert scale (1 = strongly disagree to 5 = strongly agree), evaluating five domains relevant to emergency care: Clinical Skills and Operational Ability (12 items), Emergency Response Ability (5 items), Teamwork and Communication Ability (3 items), Clinical Observation and Judgment Ability (2 items), and Competency in Emergency Tasks (1 item, rated 0–100). The Competency in Emergency Tasks item asked participants to self-assess their confidence in emergency response by rating. The NCQ’s validity was reviewed by three senior nursing supervisors, yielding a Content Validity Index (CVI) of 0.81, indicating good validity. Internal consistency was assessed in a pilot group, with Cronbach’s alpha values above 0.80 across subscales.

#### Learning Satisfaction Scale

This tool assessed participants’ satisfaction with the simulation-based or traditional training, focusing on perceived effectiveness, clarity of instruction, and overall satisfaction, using a 5-point Likert scale. It was used for feedback and quality improvement purposes.

OSCE Stations: Three structured OSCE stations were developed to evaluate real-time emergency response performance under standardized, high-fidelity scenarios, assessing specific technical and cognitive skills: Station A (Airway Management and Cardiopulmonary Resuscitation), Station B (ECG Interpretation and Defibrillator Use), and Station C (Emergency Documentation). Skills were objectively assessed during simulation stations using a structured rubric (0–2 points per skill: 2 = fully achieved, 1 = partially achieved, 0 = not achieved) to evaluate clinical performance and decision-making under pressure. The OSCE served as both a formative and summative assessment of skill acquisition.

### Procedure

The study was conducted in three main stages: pre-intervention assessment, intervention implementation, and post-intervention evaluation, spanning from October 2023 to January 2024. A pre-test was administered in October 2023, one month before the intervention, to collect demographic data, an emergency knowledge questionnaire, and self-assessments of emergency skills and confidence. The experimental group received a simulation-based intervention on November 27, 2023, delivered in-person using an OSCE format. The intervention consisted of three simulation stations designed to target core emergency care competencies: Airway Management and CPR, ECG Interpretation and Defibrillator Use, and Emergency Documentation. Each station lasted 10 minutes: 2 minutes for case reading, 6 minutes for skill execution, and 2 minutes for feedback. Trained evaluators assessed clinical and problem-solving abilities using a structured scoring rubric (Table 1).

The control group received traditional classroom-based emergency training during the same period, consisting of lectures covering the same topics (airway management, ECG interpretation, defibrillator use, and emergency documentation) without practical simulation components. One month after the intervention (January 2024), both groups completed a post-test that included the same questionnaires and assessments as the pre-test to assess changes in emergency skills, knowledge, and confidence. The intervention timeline included pre-test data collection in October 2023, the simulation or traditional training on November 27, 2023, and post-test evaluation in January 2024, enabling within-group and between-group comparisons to evaluate the intervention’s effectiveness.

#### Data analysis

Data analyses were performed using SPSS version 29.0. Data cleaning involved removing incomplete responses, and three dropouts were excluded from the final analysis. Demographic information was summarized using descriptive statistics, with categorical variables reported as frequencies. Chi-square tests were used for categorical variables to compare baseline characteristics between groups. Independent t-tests were conducted pre- and post-intervention to assess between-group differences, evaluating potential selection bias. Paired t-tests assessed within-group changes (pre- to post-intervention) in both groups. A significance threshold of p < .05 was used, with a 95% confidence interval. Normality assumptions for t-tests were checked using SPSS, confirming the data met parametric test requirements. In addition, this study calculated effect sizes (Cohen’s d) to assess the practical significance of group differences. The interpretation followed the threshold values proposed by Cohen (1988), namely d = .2 (small), .5 (medium), and .8 (large). All subsequent analyses of effect sizes in this paper were based on these criteria.^15^

## Results

### Participant Characteristics and Group Homogeneity

Participants in the experimental group (n = 32) were predominantly aged 20–23 years (n = 20) or 24–27 years (n = 12), with none over 28, while the control group (n = 35) included 21 aged 20–23, 13 aged 24–27, and 1 over 28. Most participants in both groups were women (10 males and 22 females in the experimental group; 9 males and 26 females in the control group). Work experience in the experimental group was mostly 1–12 months (n = 24) and 8 at 13–23 months, compared to 17 and 18 in the control group, respectively. CPR frequency in the past year for the experimental group showed 16 with none, 10 with 1–5, 4 with 6–10, and 2 with over 11, versus 13, 19, 1, and 2 in the control group.

**Table 1:** Description of the three OSCE stations for evaluating the emergency response capabilities of critical care nursing staff

Station	Description of Competence	Task	Skills tested
A	Airway Management and CPR	Perform airway management and chest compressions	1. Correct BVM operation2. Proper ventilation rate3. Compression speed/depth4. 5 cycles or 2-minute switching
B	ECG Interpretation and Defibrillator Use	Interpret EKG rhythm and operate defibrillator	1. Apply EKG electrodes2. Identify/report EKG rhythm3. Operate defibrillator
C	Emergency Documentation	Document emergency records	Complete documentation based on patient situation

**Table 2:** Baseline characteristics and homogeneity test between experimental and control groups (N = 67)

Variable		Experimental Group (n=32)	Control Group (n=35)	χ^2^(df, N=67)	p
Age (years)	20 - 23	20	21	.63(2)	.429
	24 - 27	12	13		
	>28	0	1		
Work Experience (months)	1 – 12	24	17	5.53(1)	.063
	13 – 23	8	18		
Actual Number of CPRs in the Past Year	0	16	13	4.77(3)	.189
	1 – 5	10	19		
	6 – 10	4	1		
	>11	2	2		
Gender	Male	10	9	.25(1)	.616
	Female	22	26		
Work Unit	Medical ICU	12	14	.08(2)	.960
	Surgical ICU	12	16		
	Emergency Room	8	5		
Education	Technology College	14	14	.11(2)	.947
	University	17	20		
	Master’s degree	1	1		
ACLS Certification	Yes	28	30	.20(1)	.830
	No	4	5		

Note: Data are presented as number of participants (n). Chi-square tests of independence were used to assess group homogeneity. p < .05 indicates statistical significance.

Chi-square tests of independence showed no significant associations between groups for baseline characteristics, including age, χ²(2, N = 67) = .63, p = .429; work experience, χ²(1, N = 67) = 5.53, p = .063; CPR frequency, χ²(3, N = 67) = 4.77, p = .189; gender, χ²(1, N = 67) = .25, p = .616; unit of work, χ²(2, N = 67) = .08, p = .960; level of education, χ²(2, N = 67) = .11, p = .947; and ACLS certification, χ²(1, N = 67) = .20, p = .830 (Table 2). These results confirm high homogeneity between the experimental and control groups, minimizing selection bias.

### Comparisons Between Groups

Independent-samples t-tests revealed no significant differences between the experimental and control groups in rescue knowledge, skills, or confidence at pre-test or post-test (Table 3).

Rescue Knowledge: No significant differences were observed at pre-test, t_(__65)_ = .79, p = .429, or post-test, t_(__65)_ = .01, p = .994, d = .00 (no effect).

Rescue Skills: No significant differences were found at pre-test, t_(__65)_ = .49, p = .624, or post-test, t_(__65)_ = 1.29, p = .201, d = .33 (small effect).

Rescue Confidence: No significant differences were observed at pre-test, t_(__65)_ = .92, p = .357, or post-test, t_(__65) _= 1.20, p = .233, d = .30 (small effect).

These findings indicate that simulation-based training did not significantly outperform traditional training in the measured outcomes.

### Comparisons Within-Group

Experimental Group: Paired-samples t-tests showed significant improvements in rescue skills from pre-test (M = 79.2, SD = 12.3) to post-test (M = 84.1, SD = 10.9), t_(31)_ = 1.92, p = .016, d = .34 (small effect), and rescue confidence from pre-test (M = 66.4, SD = 13.1) to post-test (M = 71.2, SD = 10.9), t_(31)_ = 2.92, p = .004, d = .40 (small effect). Rescue knowledge showed no significant change, t_(__31)_ = .75, p = .457, d = .15 (no effect).

Control Group: A paired-samples t-test indicated a significant improvement in rescue confidence from pre-test (M = 62.8, SD = 17.9) to post-test (M = 67.8, SD = 11.6), t_(__34) _= 2.24, p = .027, d = .33 (small effect). No significant changes were observed for rescue skills, t_(__34) _= 1.21, p = .175, d = .20 (small effect), or rescue knowledge, t_(__34)_ = 1.92, p = .060, d = .34 (small effect).

These results suggest that simulation-based training significantly enhanced skills and confidence in the experimental group, while the control group showed improvement primarily in confidence.

#### Effect Sizes

Effect sizes (Cohen’s d) were calculated to evaluate the practical significance of both within-group (pre-test to post-test) and between-group (post-test) comparisons (Table 4). In the experimental group, simulation-based training demonstrated a small effect on rescue skills (d = .34) and on rescue confidence (d = .40), according to the thresholds proposed by Cohen (1988): small (d = .2), medium (d = .5), and large (d = .8). The control group exhibited smaller effects, particularly for rescue skills (d = .20). Between-group comparisons at post-test indicated small effects favoring the experimental group for both rescue skills (d = .33) and confidence (d = .30), suggesting a slight but non-significant advantage associated with simulation-based training.

### OSCE Performance in the Experimental Group

The experimental group’s performance across three OSCE stations was evaluated to assess skill acquisition (Table 5). Station 1 (Airway Management and CPR) had the highest average score (M = 82.3, SD = 9.8), with scores ranging from 59.1 to 100. Station 2 (ECG Interpretation and Defibrillator Use) had an average score of 69.8 (SD = 14.2), with scores from 38.9 to 88.9. Station 3 (Emergency Documentation) showed the lowest average score (M = 60.0, SD = 20.0), with the widest range (22.2 to 94.4), indicating variability in documentation skills.

## Discussion

This study examined the effect of simulation-based emergency training on the emergency skills, knowledge, and confidence of critical care nurses with less than two years of experience. Baseline characteristics, including sex, age, work experience, exposure to CPR, and previous emergency training, were not significantly different between the experimental and control groups. This homogeneity is consistent with the findings of previous studies, ensuring comparability between groups and minimizing selection bias.^7^

Although emergency knowledge scores increased after the simulation-based training, the improvement was not statistically significant (d = .15). This contrasts with the findings of Roh and colleagues,^16^ where simulation-based learning significantly improved knowledge acquisition. This inconsistency may be due to differences in instructional design, participant participation, or other contextual factors, such as variability in teaching methods or the educational backgrounds of participants.

The experimental and control groups both showed improvements in emergency knowledge, skills, and confidence scores after the intervention, with no significant differences between the groups.^7^^,^^17^ These findings suggest that simulation-based training does not necessarily outperform traditional clinical teaching but serves as a valuable complement, particularly in enhancing practical skills and confidence under structured, interactive conditions. The significant within-group improvements in the experimental group (skills: d = .34, confidence: d = .40) highlight simulation’s role in providing a safe environment for hands-on practice, which is critical for novice nurses in high-pressure settings.

**Table 3:** Pre- and post-test comparisons of rescue knowledge, skills, and confidence between groups (N = 67)

Variable	Experimental Group (*n*=32)			Control Group (*n*=35)			Experimental vs Control Group	
	Pre-test Mean (SD)	Post-test Mean (SD)	Pre-test vs Post-test p	Pre-test Mean (SD)	Post-test Mean (SD)	Pre-test vs Post-test p	Pre-test p	Post-test p
Rescue Knowledge	63.4 (19.9)	66.3 (18.2)	.457	59.7 (18.3)	66.3 (20.9)	.060	.429	.994
Rescue Skills	79.2 (12.3)	84.1 (10.9)	.016*	77.7 (13.7)	80.5 (11.7)	.175	.624	.201
Rescue Confidence	66.4 (13.1)	71.2 (10.9)	.004**	62.8 (17.9)	67.8 (11.6)	.027*	.357	.233

Note: M = Mean, SD = Standard Deviation. Paired-samples t-tests were used for within-group comparisons; independent-samples t-tests were used for between-group comparisons. *p < .05, **p < .01.

**Table 4:** Effect sizes (Cohen’s d) for within-group and between-group comparisons

Variable	Group	Pre-TestMean (SD)	Post-TestMean (SD)	Within-Groupp-value	Within-GroupCohen’s d	Between-Groupp-value(Post-Test)	Between-Group Cohen’s d (Post-Test)
Rescue Knowledge	Experimental (n=32)	63.4 (19.9)	66.3 (18.2)	.457	.15 (No effect)	.994	.00 (No effect)
	Control(n=35)	59.7 (18.3)	66.3 (20.9)	.060	.34 (Small)		
Rescue Skills	Experimental (n=32)	79.2 (12.3)	84.1 (10.9)	.016*	.34 (Small)	.201	.33 (Small)
	Control(n=35)	77.7 (13.7)	80.5 (11.7)	.175	.20 (Small)		
Rescue Confidence	Experimental (n=32)	66.4 (13.1)	71.2 (10.9)	.004**	.40 (Small)	.233	.30 (Small)
	Control(n=35)	62.8 (17.9)	67.8 (11.6)	.027*	.33 (Small)		

Note: Within-group d for skills used raw data with paired correlations; for knowledge and confidence, approximate d values used pooled SDs due to unavailable raw data correlations. Between-group d used post-test scores. Effect sizes are interpreted as small (d = .20), medium (d = .50), or large (d = .80), per Cohen (1988). *p < .05, **p < .01.

**Table 5:** OSCE scoring statistics for the experimental group (n=32)

Station	Examination Topic	Highest Score	Lowest Score	SD	Average Score
Station 1	Airway Management and CPR	100	59.1	9.8	82.3
Station 2	ECG Interpretation and Defibrillator Use	88.9	38.9	14.2	69.8
Station 3	Emergency Documentation	94.4	22.2	20.0	60.0

Note: Scores are reported on a 0–100 scale, derived from the standardized rubric (0–2 points per skill) and scaled for consistency.

### Limitations

This study had several limitations that should be considered. First, it was conducted at a single medical center, limiting the generalizability of the findings to other healthcare settings. Second, the sample size was relatively small (n = 67), which may have reduced the statistical power to detect subtle differences between groups, particularly for between-group comparisons. Third, the study focused exclusively on critical care nurses with less than two years of experience, excluding the perspectives and performance of more experienced nurses, which may differ. Fourth, the reliance on self-report assessments for knowledge and confidence introduces the risk of response bias, as participants may overestimate or underestimate their abilities. While the OSCE provided objective skill evaluations, future studies should incorporate additional objective measures, such as direct observation or patient outcome metrics, to enhance reliability. Finally, the quasi-experimental design, while practical in a clinical setting, lacks the rigor of randomization, potentially introducing selection bias despite baseline homogeneity.

## Conclusions

This study compared the effectiveness of simulation-based and clinical-based emergency training in improving emergency knowledge, skills, and confidence among critical care nurses with less than two years of experience. Both training methods produced similar improvements in all measured outcomes, with no significant differences between the groups. These results suggest that traditional clinical training can effectively promote independent learning and skill acquisition in real-world environments, while simulation-based training offers a structured, experiential approach that significantly enhances skills and confidence (d = .34 and .40, respectively). Integrating simulation-based training with traditional clinical experience can provide a comprehensive strategy to optimize the development of critical care competencies in novice nurses, ultimately improving patient safety and nurse retention. Future research should involve multi-center studies with larger, more diverse samples, incorporate objective performance measures, and explore the long-term impact of combined training approaches on clinical outcomes.

The findings have significant implications for nursing education and clinical practice. By demonstrating that simulation-based training enhances skills and confidence, this study supports its integration into nursing curricula to better prepare novice critical care nurses for high-stakes emergency scenarios. This approach can improve patient safety by equipping nurses with practical competencies and fostering their ability to respond effectively under pressure. Furthermore, increased confidence may enhance job satisfaction and retention rates among novice nurses, addressing critical staffing shortages in critical care settings. Educators and hospital administrators should consider adopting hybrid training models that combine simulation with traditional methods to maximize learning outcomes.

### Acknowledgments

We express our heartfelt gratitude to the Changhua Christian Hospital for providing financial support for this study. Special thanks are extended to the critical care nursing team for their invaluable assistance and collaboration during this study. Their dedication and expertise were crucial to the successful implementation of this study.

### Conflict of Interest

The author declares that there is no conflict of interest.
